# Efficiency of Multiparticulate Delivery Systems Loaded with Flufenamic Acid Designed for Burn Wound Healing Applications

**DOI:** 10.1155/2019/4513108

**Published:** 2019-02-05

**Authors:** Sorina Dinescu, Simona Rebeca Ignat, Andreea Daniela Lazar, Ștefania Marin, Elena Danilă, Maria M. Marin, Denisa Ioana Udeanu, Mihaela Violeta Ghica, Mădălina G. Albu-Kaya, Marieta Costache

**Affiliations:** ^1^Department of Biochemistry and Molecular Biology, University of Bucharest, Bucharest 050095, Romania; ^2^Department of Collagen Research, Division of Leather and Footwear Research Institute, The National Research & Development Institute for Textiles and Leather, Bucharest 031215, Romania; ^3^Faculty of Applied Sciences Politehnica University of Bucharest, Bucharest 060042, Romania; ^4^Faculty of Applied Chemistry and Material Science, Politehnica University of Bucharest, Bucharest 011061, Romania; ^5^Department of Clinical Laboratory and Food Safety, Faculty of Pharmacy, “Carol Davila” University of Medicine and Pharmacy, Bucharest 020956, Romania; ^6^Department of Physical and Colloidal Chemistry, Faculty of Pharmacy, “Carol Davila” University of Medicine and Pharmacy, Bucharest 020956, Romania

## Abstract

Burns are soft tissue injuries that require particular care for wound healing. Current tissue engineering approaches are aimed at identifying the most efficient treatment combinations to restore the tissue properties and function by using adapted scaffolds or delivery platforms for tissue repair and regeneration by triggering molecules. To reduce the inflammation associated with skin burns, the addition of an anti-inflammatory factor in these scaffolds would greatly increase the quality of the therapy. Therefore, this study is aimed at obtaining and validating a novel multiparticulate system based on a collagen matrix with controlled delivery of flufenamic acid anti-inflammatory drug for burn wound healing applications. In this work, we have characterized the properties and biocompatibility of these multiparticulate drug delivery systems (MDDS) and we have demonstrated their efficiency against burns and soft tissue lesions, particularly when the drug was microencapsulated, and thus with a controlled release. This study contributes to the advancement in therapy of burns and burn wound healing applications.

## 1. Introduction

Skin burns are tissue injuries generally caused by heat due to the contact with boiling liquids (scalds), hot solids, or flames. According to the WHO report in 2018, 180,000 deaths are estimated to occur annually worldwide with a higher rate in low- and middle-income countries [1].

The skin has critical roles in maintaining the body fluid homeostasis and thermoregulation, being considered the body's largest and active immune organ involved in the first defense barrier. Thermal burns are complex processes that demand careful guided treatment to promote wound healing, reestablishing the immune barrier, and fast tissue regeneration with minimum scaring.

The healing process of the thermal burns comprises four overlapping phases including an initial phase of tissue homeostasis activated in the first couple of minutes after injury followed by posttraumatic inflammation and, in a couple of days, by the proliferation and skin remodeling phases [2].

The first two phases of activated postinjury are critical for the wound healing evolution and scaring. Among the processes activated immediately after injury are immune activation and platelet aggregation with blood clotting in order to protect the affected area and supply the scaffold*-*like matrix for the migration of cells responsible for skin integrity like dermal fibroblasts, keratinocytes, leucocytes, and endothelial cells. The posttraumatic inflammatory phase is generally initiated by some cytokines such as tumor necrosis factor (TNF-*α*), interleukin-1 (IL-1), and interleukin-6 (IL-6) to promote local inflammation with neutrophil extravasation and macrophage activation. An impaired inflammatory process prolonged local pain and stimulates the cellular secretion of extracellular matrix with consequences on thick scars [2, 3].

Dressings like gauzes and hydrogels are formulations that can be made from natural (alginate, collagen, and cellulose) and synthetic (polyvinyl alcohol, caprolactone, and polylactic acid) polymers: foams and hydrocolloids by carboxymethyl cellulose, transparent films by polyurethane. The research is focused to develop combinations of polymers as candidates to obtain ideal dressings to stimulate the wound healing [4–7].

Because of their high biocompatibility and similarity to the extracellular matrix (ECM), the natural polymers are widely used in wound dressing preparation [8–11]. Collagen presents low antigenicity and low inflammatory properties, good biocompatibility, water-absorbing ability, and haemostatic properties and has the ability to promote cell attachment [12–15]. Pure collagen may not satisfy all requirements that an ideal wound dressing should have; therefore, it needs to be combined with some anti-inflammatory agents, like drugs or natural plant extracts. Providing the biological scaffold like collagen and administrating a short-term anti-inflammatory agent are helpful for cellular migration to promote the skin regeneration, decrease the local pain, and maintain a balanced inflammatory level favorable in a long-term cicatrizing process [2, 3, 16].

So far, only few biopolymer-based wound dressings with nonsteroidal anti-inflammatory drugs (NSAIDs) were developed [17–20].

A limitation of some of these formulations is that the rapid release effect is too pronounced during the first hours, with the risk of an important amount of drug to be released before the inflammation is controlled [17, 21–23].

In this respect, the vehicle in the drug delivery sciences is a critical quality attribute which needs special attention for a tailor-made design to rationalize the formulation development. Thus, new drug delivery systems have to be developed in order to ensure a proper delivery of the anti-inflammatory drug from spongious matrices using different techniques that allow a gradual degradation of collagen support and a slow release of drug.

In this context, to reduce the burst release effect and to provide a controlled drug release, we propose in this study the microencapsulation of an anti-inflammatory drug in biodegradable polymeric supports which results in topical multiparticulate drug delivery systems (MDDS).

Based on our previous studies concerning some designed collagen-dextran-flufenamic acid collagen sponges, the aim of this work was (1) to obtain multiparticulate systems based on the collagen matrix with controlled delivery of an anti-inflammatory drug (flufenamic acid was selected as a model NSAID) embedded in gelatin-alginate-carboxymethyl cellulose microcapsules, (2) to investigate their biocompatibility for future wound healing applications, and (3) to evaluate the release mechanism, degradation rate, absorption capacity, and efficiency against *in vitro* inflammation modelling *in vivo* behavior. The preclinical *in vivo* studies involving animal models are very important and frequently used following the *in vitro* studies to evaluate the efficacy of a novel product designed for burn healing bringing substantial advancements in the therapy of burns [2, 3, 16, 24–26].

## 2. Materials and Methods

### 2.1. Achievement of MDDS

#### 2.1.1. Materials

The type I fibrillar collagen gel (Col) having an initial concentration of 1.92% (*w*/*v*) and gelatin (Gel) with an initial concentration of 2.5% were extracted from calf hide using technology currently available in Collagen Department from Research-Development Textile Leather National Institute Division Leather and Footwear Research Institute [25]. The collagen gel, dextran sulfate sodium salt (Dex) with a molecular weight of 40,000 (Alfa Aesar, Germany), and flufenamic acid (FA) (MP Biomedicals, USA) were raw materials for matrix (spongious form) preparation. Gelatin, sodium carboxymethyl cellulose (CMCNa) (Fluka), and alginic acid sodium salt from brown algae (Alg) (Sigma-Aldrich, Germany) were chosen to prepare microcapsules. Glutaraldehyde (GA) from Sigma-Aldrich (Germany) has been used for matrix cross-linking, and calcium chloride (CaCl_2_) from Merck has been used for microcapsule cross-linking. Sodium hydroxide from Merck (Germany) was of analytical grade, and the water was distilled.

#### 2.1.2. Preparation of Microcapsules

1 g of sodium alginate, 0.25 g CMCNa, and 2.5 g gelatin were solubilized in distilled water, and 2.4 g FA previously solubilized in 1 M NaOH solution was added and homogenized together in distilled water, at 60°C, to obtain 100 ml gel for microcapsule preparation. The gel was dropped with a syringe into a 1 M CaCl_2_ solution for cross-linking, and spherical capsules were obtained.

#### 2.1.3. Preparation of Matrices

The previously obtained microcapsules (MC) were embedded in the composite gel consisting in 0.8% collagen (Col), 1.2% dextran (Dex) reported to collagen (which means 0.96 g in 100 ml gel), and 0.5% flufenamic acid (FA) reported to collagen (which means 0.4 g in 100 ml gel) and then cross-linked with 0.006% glutaraldehyde (GA) reported to collagen (which means 0.0048 g in 100 ml gel). All the samples consist in the same concentration of collagen, dextran, and crosslinking agent, the difference between them being given by FA concentration and microcapsule amount: M1 consisting in FA and no microcapsules, M2 consisting in 30 g microcapsules and 70 g composite gel without FA, M3 consisting in 15 g microcapsules and 85 g composite gel without FA, M4 consisting in 30 g microcapsules and 85 g composite gel with FA, and M5 consisting in 15 g microcapsules and 85 g composite gel with FA.

**Figure 1:**
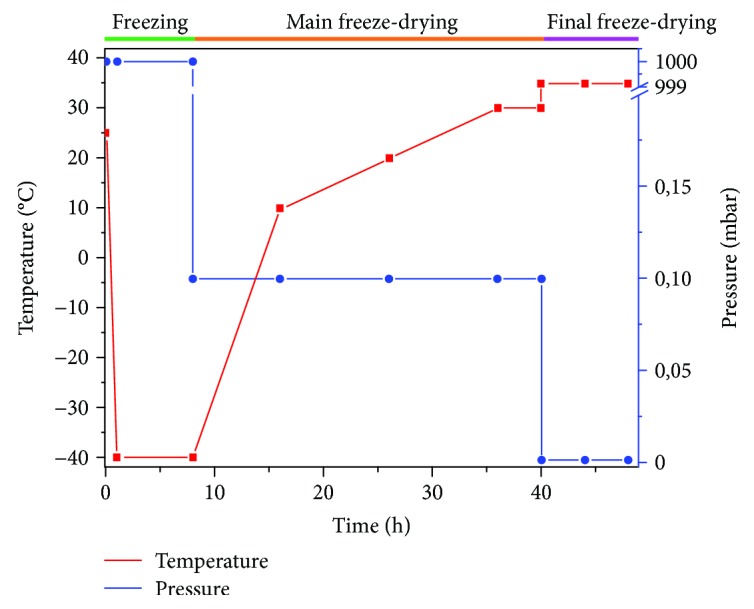
Graph chart of the freeze-drying process.

The gel compositions were cast in glass dishes (5 cm diameter and 0.5 cm height) and kept at 4°C for 24 hours for cross-linking. Then they were freeze-dried (48 hours) as we present in Figure 1, in order to obtain porous scaffolds, as follows: cooling to −40°C (4 h), keeping up for 8 h, then freeze-dried at −40°C and 0.12 mbar for 8 hours; then heating to +20°C at a rate of 3°C/hour (10 h) at 0.12 mbar, then heating (10 h) to 30°C, other 4 h at 30°C and the same pressure; and finally freeze-dried at +35°C at 0.01 mbar for 8 hours, using the Christ Model Delta 2–24 LSC freeze-dryer (Germany).

The matrices M1 ÷ M5 in a spongious form were obtained by lyophilization as multiparticulate drug delivery systems (MDDS) and tested by water uptake and enzymatic degradation.

#### 2.1.4. Water Uptake of MDDS

The water uptake capacity was carried out using phosphate buffer pH 7.4 as the immersion medium (*n* = 3) and calculated using the previously described methods [17]. Briefly, the samples were first immersed in PBS at 370°C. At scheduled time intervals, the samples were withdrawn, wiped (to remove the surface water), and weighed. The water uptake ability was monitored using the following equation:

(1) $$\begin{array}{c} \text{Water uptake}=\frac{\left(W_{t}-W_{0}\right)}{W_{0}}, \end{array}$$

where *W*_0_ is the dried sponge weight at the initial time and *W*_*t*_ is the sponge weight after immersion at time *t* [17, 26].

#### 2.1.5. Enzymatic Degradation of MDDS

*In vitro* enzymatic degradation of MDDS sponges by collagenase was also investigated by monitoring the mass loss of samples as a function of exposure time to a collagenase solution according to a procedure described in the literature [17, 26]. Pieces of collagen scaffolds (1 cm in diameter) were accurately weighed (wet weight without excess of water), placed in a solution of PBS and collagenase (1 *μ*g/ml) at pH 7.4, and incubated at 37°C. At regular intervals, the swollen sponges were removed from the collagenase solution, wiped, and weighed. The resistance to enzymatic degradation was determined through a weight loss parameter computed using the following equation:

(2) $$\begin{array}{c} \%\text{weight loss}=\frac{W_{i}-W_{t}}{W_{i}}\times 100, \end{array}$$

where *W*_*i*_ is the sponge initial weight and *W*_*t*_ is the weight of the samples after time *t* [17, 26]. Each biodegradation experiment was repeated 3 times. The final percentage of biodegradation was calculated as the average values.

### 2.2. *In Vitro* Drug Release Study and Data Modelling

The studies of FA release from the collagen sponges incorporating the drug in various forms (free form, free and encapsulated form, and encapsulated form in spongious matrices) were carried out using a dissolution equipment in conjunction with paddle stirrers (Esadissolver), as previously reported [16]. Briefly, the sponge samples were fixed in a transdermal sandwich device and then immersed in apparatus dissolution vessels. The kinetic studies were performed at 37°C ± 0.5°C with a rotational speed of 50 rpm. The release medium was a phosphate buffer solution of pH 7.4. At predetermined time intervals, samples of 5 ml were collected from the receiving medium and replaced with an equal volume of fresh phosphate buffer solution, kept at 37°C ± 0.5°C, to maintain a constant volume in the release vessel. The concentration of FA was spectrophotometrically assessed (Perkin-Elmer UV-vis spectrophotometer) using the standard curve (*A*_1%_^1 cm^ = 534) determined at *λ*_masx_ = 288 nm, and the cumulative released drug percentage was determined.

### 2.3. *In Vitro* Assessment of Material Biocompatibility

A culture of human adipose-derived stem cells (hASCs) was obtained (Gibco, Thermo Fisher, USA) and maintained in standard conditions (370°C, 5% CO_2_). The cells were cultured in Dulbecco's Modified Eagle's Medium (DMEM) with 10% fetal bovine serum (FBS) and 1% antibiotic antimycotic solution (Sigma Aldrich, Germany). The cells were seeded and allowed to distribute inside the 3D materials, and the biocompatibility of the materials was assessed 3 and 6 days after cell seeding. All the tests were realized in triplicate.

The viability and the proliferation rate of the cells in contact with the materials were evaluated by the MTT test (Sigma-Aldrich, Germany). The materials seeded with cells were washed with PBS before incubation with MTT solution 1 mg/ml at 370°C for 4 hours. The formazan produced by the metabolic active cells was solubilized in isopropanol, and its absorbance was measured by spectrophotometry at 550 nm. The values obtained were proportional with the number of viable cells in the materials.

The levels of cytotoxicity induced by the materials were obtained by LDH assay (TOX7-1KT, Sigma-Aldrich, Germany). The LDH released by the cells that lost membrane integrity was quantified by spectrophotometry, measuring the absorbance at 490 nm. The values obtained were proportional with the amount of cells that died in contact with the materials.

For the qualitative evaluation of the behavior of the cells in contact with the materials, the dead and live cells were visualized by fluorescence microscopy. The solution with calcein and ethidium homodimer obtained from the Live Dead kit (ThermoFisher, USA) allowed staining and visualization of the live cells in green fluorescence and dead cells in red fluorescence.

### 2.4. *In Vitro* Evaluation of the Anti-inflammatory Potential of the MDDS

In order to evaluate the anti-inflammatory properties of the M1—M5 systems, a culture of murine macrophages from RAW 264.7 cell line was obtained in standard conditions. Twenty-four hours before putting the cells in contact with the materials, the macrophages were activated with 1 *μ*g/ml lipopolysaccharide (LPS) (Sigma-Aldrich, Germany). The levels of proinflammatory mediators in response to the FA anti-inflammatory properties were evaluated 48 hours after cell seeding both at gene and protein levels of expression. A control sample of activated macrophages exposed to no other treatment was considered positive reference in analysis.

### 2.5. Screening of Proinflammatory Mediator Levels of Expression Multiplex Assay

The levels of IL6, TNF*α*, MCP1, and RANTES cytokines and chemokines were measured from cell culture media 48 hours after induced macrophages were put in contact with the M1—M5 systems, using a customized magnetic bead-based multiplex assay (Merck Millipore, Germany) and detection with a Luminex 200 system. Data was analyzed using xPONENT software.

### 2.6. Gene Expression Evaluation by qPCR

IL6 gene expression was analyzed by qPCR, considering a comparison between the levels of IL6 in murine macrophages exposed to M1—M5 materials and the level of IL6 in LPS-induced macrophages. Briefly, the total RNA from RAW cells was isolated using Trizol reagent (ThermoFisher, USA), according to the manufacturer's protocols and tested for integrity on a BioAnalyzer 2100 (Agilent Technologies, Germany). One microgram of total cellular RNA was reverse transcribed to the corresponding cDNA using an iScript cDNA Synthesis kit (BioRad, USA). Sequences of IL6 gene-specific primers used in q-PCR assay are 5′-AGTTGCCTTCTTGGGACTGA-3′ and 5′-TCCACGATTTCCCAGAGAAC-3′. The relative gene expressions of IL6 was evaluated by qPCR on a LightCycler 2.0 system (Roche, Germany) and was normalized to GAPDH reference gene.

### 2.7. Cytokine Expression Evaluation by Fluorescence Microscopy

IL6 protein expression was investigated by immunostaining and fluorescence microscopy 48 hours after induced macrophages were put in contact with M1—M5 materials. Briefly, cells in matrices were fixed with 4% paraformaldehyde for 2 hours and then permeabilized for 1 hour with a 0.1% Triton X100 solution in 2% BSA. The systems were further immunostained overnight using a primary anti-IL6 antibody (sc-1266, 1 : 100 dilution, Santa Cruz, USA) and then secondary antibody conjugated with FITC (sc-2777, 1 : 100 dilution, Santa Cruz, USA) for 1 hour at 4°C. Cell nuclei were stained with DAPI. Staining was visualized in an Olympus IX73 fluorescence microscope.

### 2.8. Animal Model of Experimentally Induced Burns

The experiment was performed on 30 Wistar rats weighing 160 ± 10 g purchased from the Animal Biobase of the “Carol Davila” University of Medicine and Pharmacy, Bucharest.

All animals used in the study were kept in standard laboratory conditions, were fed twice a day, and received water *ad libitum.* The experiment was performed in compliance with the European Communities Council Directive 2010/63/UE and Law No. 43 of the Romanian Parliament from 11.04.2014.

The animals were distributed in 7 groups of 5 individuals each as follows: group 1—M1, group 2—M2, group 3—M3, group 4—M4, group 5—M5, group 6—control (burns), and group 7—negative control (healthy animals used for hematological analyses).

The animals belonging to groups 1–6 were anesthetized with ether ethylic, and the hair was removed from the dorsal area. A special metallic device containing a 10 mm diameter disc was used for inducing the experimental wound. The device was heated in boiling physiological serum and applied on the shaved dorsal area for 15 seconds. The lesions measuring 10 mm diameter were sterilized, and the collagen scaffolds were applied and fixed with a silk plaster for groups 1–5. The control group (group 6) used in the study received the classical burn treatment by covering the wounds with sterile cotton dressing.

The surface morphology evolution of the wounds was recorded using a digital camera (Olympus SP-590UZ), and the wound diameter was measured for 17 days.

The healing process was evaluated according to the size profile of the wound as described by the following equation:

(3) $$\begin{array}{c} \text{Healing process}\%=\frac{\left(\text{wound diameter at }t=0\right)-\left(\text{wound diameter at }t\right)}{\text{wound diameter at }t=0}\times 100, \end{array}$$

where the wound size was an average measurement of the longest and shortest dimensions of the affected area [2, 3, 12, 16]. The wound was considered healed after the crust of the lesion fell off.

Any secondary effects such as inflammation or infection of the wounds, as well as any variations on the animal health status, were also monitored. In the last day of the monitoring, the animals were ethyl-ether anesthetized and slaughtered. The blood was collected in K_3_EDTA anticoagulant vacutainers for hematological tests to evaluate any possible secondary effects like inflammation or anemia induced by the treatment. A supplementary negative control group (group 7) (*n* = 5) monitored for 17 days with no experimentally induced burn or treatment was used for the comparison of the hematological data.

The blood analyses were performed using an Abacus Junior hematological equipment. The specific reagents were purchased from the Diatron company.

### 2.9. Statistical Analyses

Statistical analyses were performed using the GraphPad Prism 6 software. All data were expressed in the mean and standard deviations (SD). Normal distribution was calculated using the Kolmogorov–Smirnov test. The experimental data were evaluated using the student *t*-test and analyses of variance followed by Dunnett's multiple comparison test.

For in vitro biocompatibility tests and anti-inflammatory potential of the MDDS, a one-way ANOVA algorithm of analysis was used, followed by Bonferroni's posttest. The results were considered significant at *p* < 0.05, highly significant at *p* < 0.01, and not significant at *p* > 0.05.

## 3. Results and Discussion

### 3.1. Evaluation of Water Uptake and Resistance to Enzymatic Degradation

Water uptake of obtained matrices was investigated by gravimetric analysis in order to evaluate how the microcapsules influenced the MDDS.

**Figure 2:**
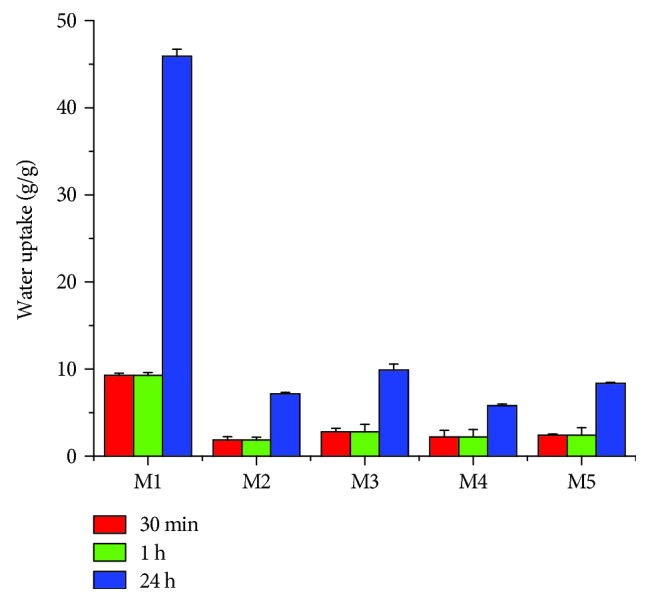
Water uptake of multiparticulate systems.

According to our results, the highest amount of water was absorbed by M1 sample, having no microcapsules in the composition (Figure 2). The samples with a low amount of microcapsules (M3 and M5) absorbed higher amount of water than samples with a higher amount of microcapsules (M2 and M4). This is because the microcapsules swell themselves in contact with water and perform a denser and compact structure of sponges with smaller porosity.

Multiparticulate systems absorbed water quickly in the first 30 min and then continued gradually, until it reached equilibrium after 24 h.

**Figure 3:**
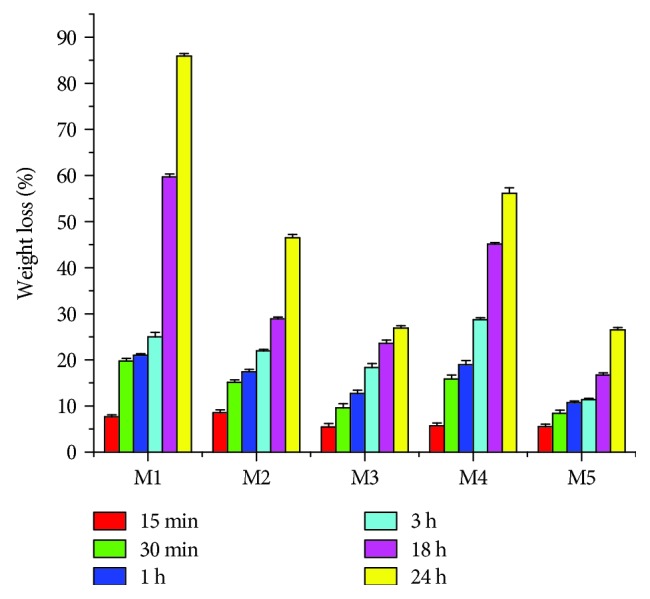
Enzymatic degradation of multiparticulate systems.

After swelling for 24 hours, the MDDS were evaluated by degradation in collagenase solution in order to simulate their *in vivo* behavior and to investigate their stability (Figure 3).

The sample without microcapsules (M1) was degraded faster than the multiparticulate systems (Figure 3), about 85% in 24 hours. The microcapsules swollen and make the sponge more resistant to degradation.

**Figure 4:**
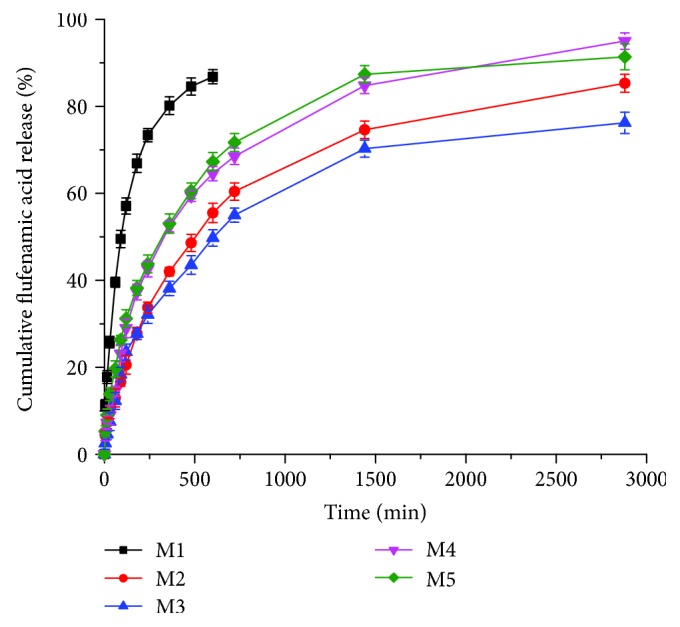
Cumulative release profiles of FA from topical spongious matrices as a function of time.

The release of FA from the designed supports is illustrated in the cumulative kinetic profiles given in Figure 4.

Correlating data from Figures 2 and 3, the water uptake ability and the resistance to enzymatic degradation of the designed systems are strongly influenced by the presence of microcapsules in the formulation, the amount of FA, and the ratio between collagen gel and microcapsules.

### 3.2. Release of FA from MDDS

Regarding the release of FA from the multiparticulate systems, a similar biphasic shape was noticed for the kinetic profiles recorded at the release in phosphate buffer pH 7.4 (Figure 4). Thus, FA was released faster in the first 60 min, followed by a slow release during several hours of experiment (up to 10 hours for the supports incorporating FA in a free form and up to 48 hours for the multiparticulate systems; FA incorporated in the spongious matrix in an encapsulated form and in a free and encapsulated form, respectively). This initial step of drug release, known as the “burst release” effect, is more evident for the support incorporating FA in a free form (M1), the drug being released in a percentage of 39.54% in comparison with the multiparticulate systems, whose released FA percentage varies between 12.25% (M3) and 19.65% (M5). After 10 hours, the released FA percentage reach 86.81%. After 48 hours, the released FA percentage varies between 76.21% (M3) and 95.01 (M4) (Table 1). The decrease of drug amount released fast in the first hour is due to a lower absorption capacity of the multiparticulate systems as a consequence of the encapsulation technique. The burst release effect reduces the pain sensation and the amount of proinflammatory mediators released at the burn wound site, while the slow and gradual release during the following hours ensured an anti-inflammatory and analgesic effect for a longer period of time needed for burn healing. Such kinetic profiles with drug biphasic release are targeted for the control of local inflammation and pain associated to a cutaneous wound because the first 12–48 hours are critical in a wound healing process.

To evaluate the mechanism of the FA release from the supports tested, various kinetic models were applied to the experimental data: Power law model (4), as well as its particular cases, Higuchi (*n* = 0.5) and Zero-order (*n* = 1).

(4) $$\begin{array}{c} \frac{m_{t}}{m_{\infty }}=k\cdot t^{n}, \end{array}$$

where *m*_*t*_/*m*_∞_ is the fractional drug released in time *t*, *k* is the kinetic constant, and *n* is the release exponent characteristic to the drug transport mechanism.

The values recorded for the correlation coefficient were higher for the Power law model (*R* > 0.97) compared to the Higuchi and Zero-order models, indicating a non-Fickian *release kinetic mechanism* of FA from the spongious matrices loaded with FA in a free form, i.e., from the multiparticulate systems, the release exponent values ranging from 0.36 to 0.41. The kinetic constant and the release exponent value characteristics to this model are given in Table 1.

**Figure 5:**
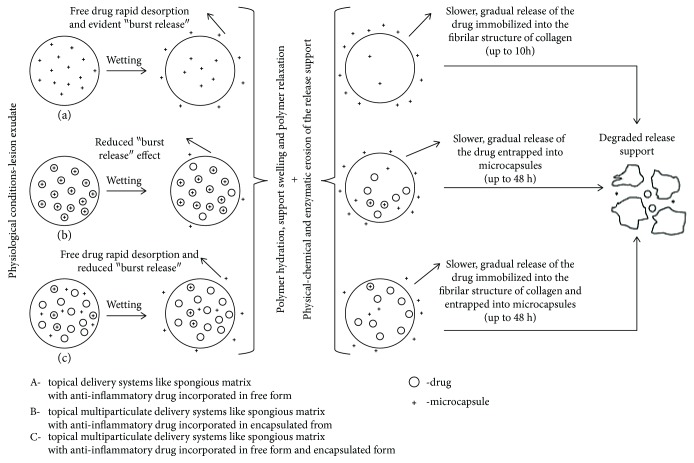
The drug kinetic release mechanism form of different topical delivery systems: (a) spongious matrix with an anti-inflammatory drug incorporated in a free form—M1; (b) multiparticulate delivery systems like the spongious matrix with an anti-inflammatory drug incorporated in an encapsulated form—M2 and M3; (c) multiparticulate delivery systems like the spongious matrix with an anti-inflammatory drug incorporated in a free form and encapsulated form—M4 and M5.

The kinetic mechanism associated to the release profiles is presented in Figure 5. Thus, an important amount of exudate can be initially found at the burn wound level, wetting the spongious matrix surface, being then absorbed in and penetrating the matrix porous structure. Upon contact between the spongious matrix and wound, a gel layer is formed favoring the diffusion of the free-form drug entrapped at and closed to the surface and released through desorption (for the matrices where FA is incorporated in free form and free and encapsulated form, respectively), explaining the FA fast release effect (“burst release” effect) in the first 60 minutes. For the multiparticulate supports, this initial drug release step is slower. The wound exudate absorption process is followed by polymeric network hydration and relaxed polymer swelling implying a slower, gradual diffusion of the free-form drug incorporated (partially immobilized in collagen fibrillary structure) and of the drug from the microcapsules entrapped in the polymer matrix during the lyophilization process, respectively, concomitantly with the much slower process of release support degradation compared to the matrices incorporating FA only in a free form.

All these physical-chemical processes are explaining the deviation from the Higuchi model, characterized by a slower drug diffusion rate in comparison with polymer relaxation rate and also from the Zero-order model for which the drug diffusion rate is higher than the polymer relaxation rate.

### 3.3. *In Vitro* Biocompatibility of the Multiparticulate Systems

Multiparticulate drug delivery systems (MDDS) were evaluated for biocompatibility in terms of cell viability and MDDS cytotoxicity.

**Figure 6:**
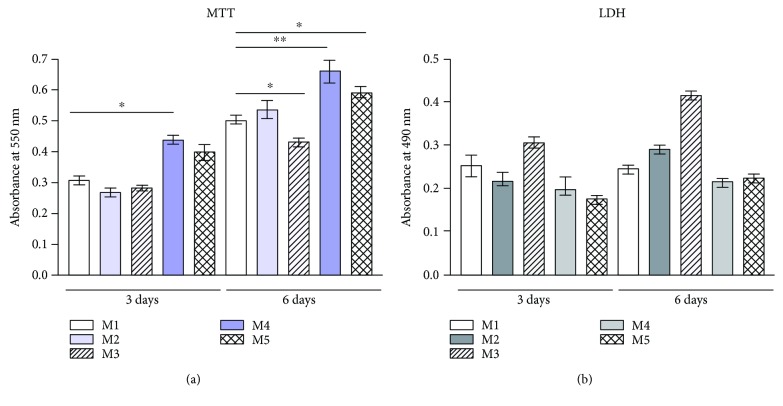
(a) Cell viability obtained after 3 and 6 days of hASCs contact with M1—M5 matrices, as shown by MTT quantitative assay. (b) Cytotoxic potential of M1-M5 matrices, as revealed by LDH assay after 3 and 6 days of culture in standard conditions.

Quantitative results obtained by MTT assay revealed an overall good biocompatibility of the materials. Three days after cell seeding in contact with the materials, a similar cell viability was found for the M2 and M3 systems, as compared to M1 material, considered as a control. However, in case of M4 and M5 MDDS having FA anti-inflammatory agent encapsulated, an increased cell viability was registered (*p* < 0.05). After 6 days of culture, a similar cell viability profile was found (Figure 6(a)). Cells cultured in contact with the M2 system revealed similar viability to those cultured in contact with M1 control, thus proving that the addition of microcapsules (MC) in the composition of the matrices did not influence the biocompatibility of the system. Interestingly, a statistically significant decrease in cell viability was observed in case of the M3 system, as compared to the control. An increase in cell viability was registered for M4 and M5 MDDS (*p* < 0.01 and *p* < 0.05, respectively), suggesting that the incorporation of FA in the microcapsules and resulted MDDS structure and properties displayed a positive effect on cell viability.

By contrast, LDH assay offered quantitative information on the cytotoxic potential of the MDDS (Figure 6(b)). After 3 days of culture, the LDH levels released in the culture media were similar for all M1-M5 matrices, suggesting a low and comparable degree of cytotoxicity for the systems. After 6 days of culture, materials exerted almost no increase in the cytotoxicity profile for M2, M4, and M5, as compared to M1 control, showing that even if the cells registered proliferation from 3 to 6 days of culture (Figure 6(a)), the cytotoxic levels remained to the same levels. A slight increase in material cytotoxicity was found for M3 after 6 days of culture, resulting in the lowest biocompatibility of this material among all tested compositions.

**Figure 7:**
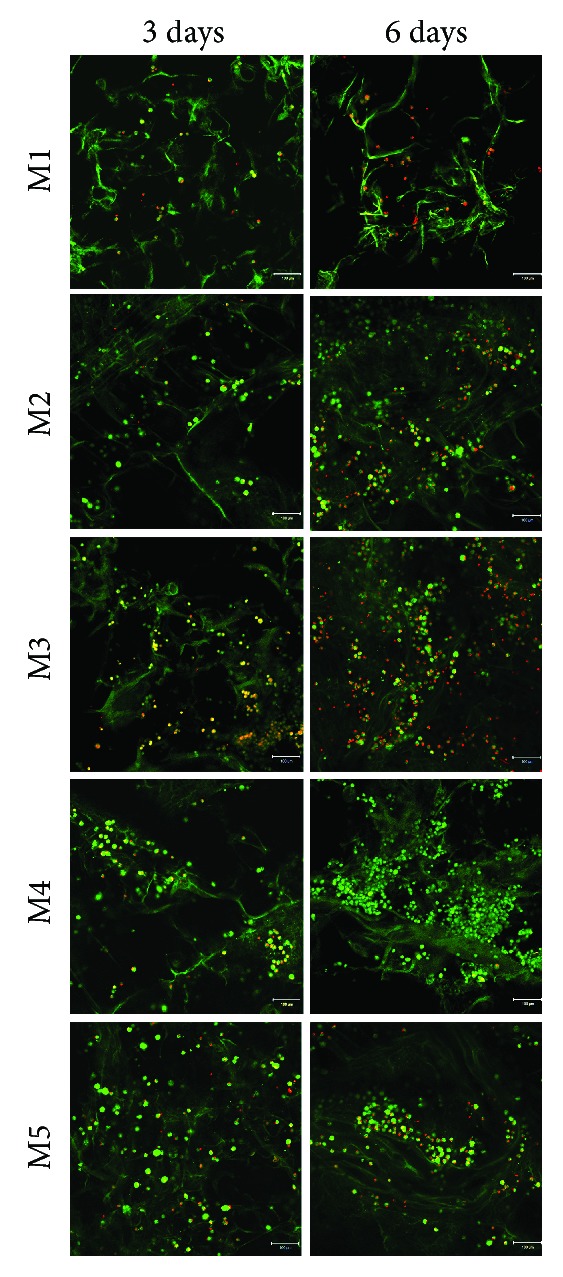
Live (green) cells and dead (red) cells visualized after 3 and 6 days of culture in the M1–M5 systems by confocal microscopy.

The visualization of both live and dead cells in the three-dimensional cell cultures was possible by Live Dead assay and visualization in confocal microscopy (Figure 7). Live Dead assay results are in accordance with the quantitative data obtained from MTT and LDH assays, revealing the most biocompatible system to be M4. All tested MDDS revealed an overall good biocompatibility, particularly the M4 and M5 systems, where a strong positive ratio between live green cells and dead red cells was observed. Additionally, the highest amount of dead cells marked by red fluorescence was found in contact with M3 composition after 6 days of culture, confirming the LDH results. Generally, a higher amount of live cells and a tendency to group was found in M4 and M5 materials, in comparison with M1 control, thus suggesting that the incorporation of FA in microcapsules favored material biocompatibility.

### 3.4. Evaluation of the Anti-inflammatory Potential of MDDS

**Figure 8:**
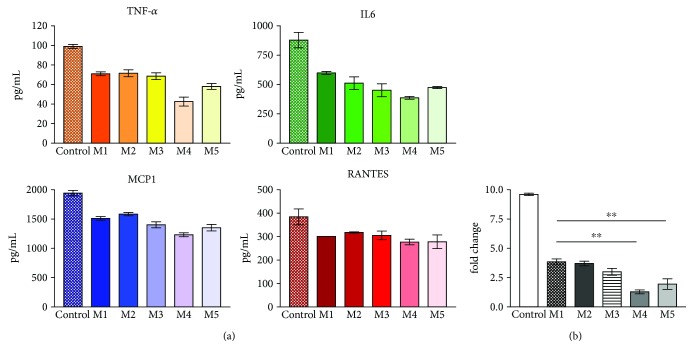
(a) Protein levels of TNF*α*, IL6, MCP1, and RANTES proinflammatory mediators found in cell culture media of activated macrophages after contact with the M1–M5 systems, as compared to the positive control. (b) IL6 gene expression in activated macrophages exposed to M1-M5, as compared to the activated control.

Screening among several cytokine and chemokine levels was performed in order to investigate the effect of FA on inflammation and the anti-inflammatory potential of the M1–M5 systems (Figure 8(a)).

Screening results indicated a clear decrease in proinflammatory cytokine and chemokine levels secreted by cells in contact with M4 and M5, as compared to M1 control and M2 and M3. This suggests that the encapsulation and gradual release of FA up to 48 hours result in a more efficient anti-inflammatory response. In particular, the lowest levels of IL6 and TNF*α* were registered for M4 composition, which contains the highest amount of microcapsules filled with FA. The levels of MCP1 and RANTES remained approximatively constant among all studied compositions, but showing a tendency to decrease also in the systems with encapsulated FA. The most significant changes were found in IL6 levels; therefore, IL6 expression was further assessed at gene level and by immunostaining.

IL6 gene level of expression was evaluated by qPCR and revealed a statistically significant lower mRNA level after activated macrophage exposure to M4 material (Figure 8(b)). The relative gene expression registered a fold change of 10 when comparing control-activated macrophages with the cells exposed to M4 material. This decrease in IL6 expression could be correlated to the higher content of microcapsules with FA in the M4 system and could be probably attributed to the gradual controlled released of the anti-inflammatory agent from the matrices. IL6 transcript level detected by qPCR is in accordance with IL6 profile of protein expression and revealed a higher anti-inflammatory efficiency of the multiparticulate drug delivery systems containing microcapsules loaded with FA than the matrices incorporating free FA in the composition.

**Figure 9:**
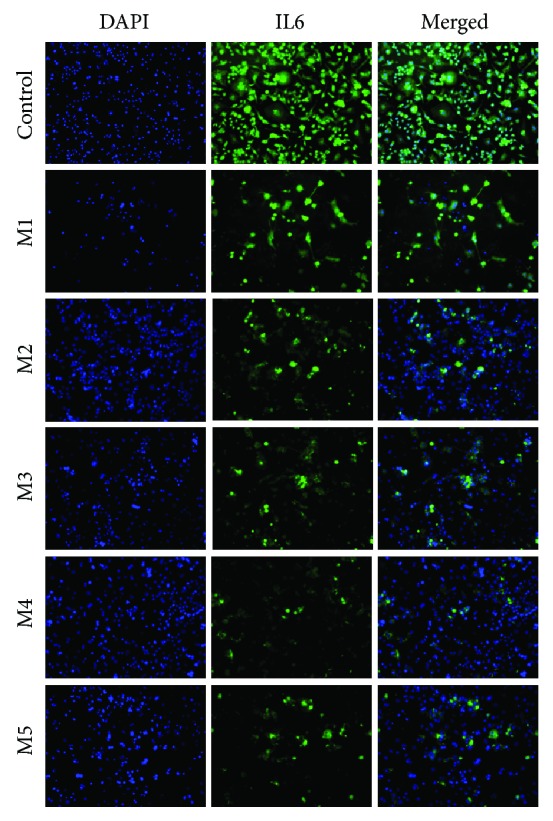
Expression of IL6 in macrophages exposed for 48 h to the M1–M5 systems, as compared to the positive control.

Immunostaining of IL6 and visualization in fluorescence microscopy (Figure 9) showed a significant decrease in IL6 level of expression in cells exposed to the M1–M5 systems, whereas all cells in positive control activated with LPS expressed IL6. Thus, it can be concluded that all analyzed materials have an anti-inflammatory effect to a lower or higher degree. The lowest IL6 expression was detected for M4 material, confirming previous gene expression results. Results obtained for M1, M2, M3, and M5 are comparable in terms of IL6 expression.

### 3.5. *In Vivo* Evaluation of MDDS Efficiency for Burn Treatment

**Figure 10:**
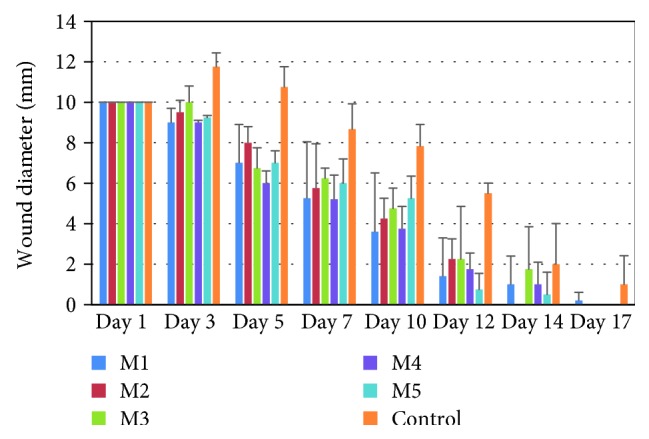
Wound diameter (mm) evolution after treatment with spongious matrices loaded with FA in experimentally induced burn to Wistar rats (bars in the graph represent standard deviation).

**Figure 11:**
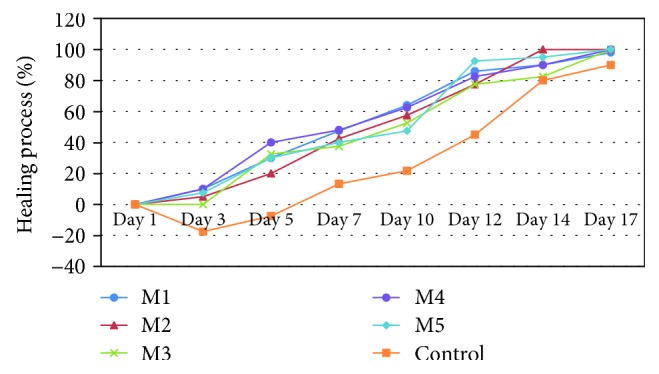
The evolution of the healing process (%) in experimentally induced burns to Wistar rats after the treatment with a biopolymeric collagen matrix loaded with an anti-inflammatory drug.

**Figure 12:**
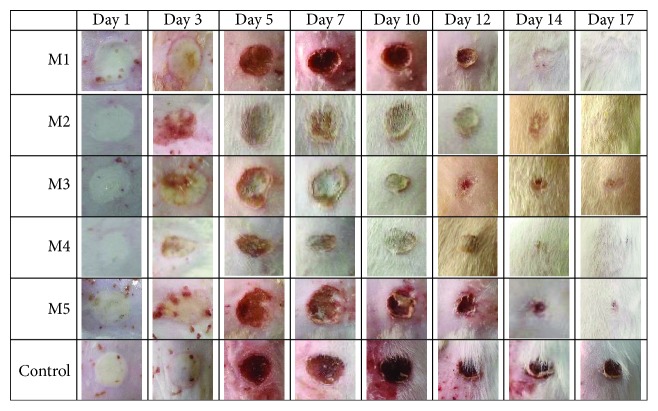
The macroscopic morphology of the experimentally induced burns to Wistar rats after the treatment with spongious collagen matrices loaded with an anti-inflammatory drug and a classical treated control group.

The evolution of the wound diameter after the treatment with collagen matrices loaded with FA incorporated in various forms is presented in Table 2 and Figure 10, and the healing process is presented in Figure 11. The macroscopic morphology of the experimentally induced burns is presented in Figure 12.

The evolution of the burn is a complex process comprising generally the following overlapping phases: an initial local homeostasis followed by inflammation, proliferation, and tissue remodeling.

The first two phases (homeostasis and inflammation) occurring in the initial 1–5 days are critical for the cicatrizing process of the wounds.

After inducing the burn on experimental animals, the local area was characterized by a white eschar, the surface skin layers (epidermis and dermis) were damaged, and during the next hours, the lesion became fully hyperemic due to erythrocyte extravasation. Several homeostatic processes are activated, like platelet aggregation, immune defense, and blood clotting.

In the first three days, the posttraumatic inflammation is generally characterized by an increased level of proinflammatory cytokines stimulating the neutrophil migration and the monocytes switching into activated macrophages [27]. Releasing of strong inflammatory signals could interfere the skin remodeling and regeneration processes, promoting the formation of thick scars by increasing the secretion of the extracellular matrix. The use of NSAIDs for a short term applied in different topic formulas decreases the local pain and favors the generation of a normal skin with minimal scarring comparing to a long-term use of with impaired wound healing [2, 16, 23].

The treatment with collagen sponges applied in case of groups 1–5 decreased the local damage in the initial homeostatic phase by supplying a scaffold-like matrix for the migration of the first-line defense cells like dermal fibroblasts, keratinocytes, leukocytes, and endothelial cells [2, 16].

Following the next days, the reepithelization process was accelerated in treated groups and was associated with decreasing wound diameters in these groups comparing to the control group (animals with experimentally induced burns without spongious matrix treatment).

The treatment with a collagen spongious matrix was proved to be efficient in the posttraumatic inflammatory phase by decreasing the wound diameter favoring the healing process in days 3, 5, and 7, a critical important period in the cicatrizing process. For 5 days, a slight inflammatory posttraumatic effect was noticed in case of the control group reflected by an increased wound diameter in comparison with all treated groups.

After 5 days (Table 2, Figure 12), the most significant decrease of the wound diameter by 40% was noticed in case of the treatment with an M4 spongious matrix followed by M3 (32.5%), M1 (30%), and M5 (30%).

All treatments significantly favored the wound healing process (*p* < 0.05) after the first week. The most significant results were noticed in case of M1 and M4 treatments with a healing process of almost 48% (*p* < 0.05).

After 14 days, most of the animals from the treated groups were completely healed with reepithelized lesions (Table 2, Figures 10–12) compared to the control group characterized by wounds with thick scars and a healing process extending the 17 days of monitoring.

The most commonly developed secondary effects after burn injuries are inflammation, infections, or anemia. The hematological analysis of some blood parameters is usually performed to identify any possible systemic effects after inducing the burns and treating new developed biopolymeric collagen sponges loaded with FA (Tables 3 and 4).

The following classical hematological parameters were used to detect the presence of anemia: red blood cell (RBC), hemoglobin (HGB), hematocrit (HCT), mean cell volume (MCV), mean cell hemoglobin (MCH), mean cell hemoglobin concentration (MCHC), and red blood cell distribution width (RDW).

The hematological parameters correlated with anemia or other erythrocyte pathologies presented no significant variation in case of treated groups (M1–M5) and control group of animals with experimentally induced burns compared to the negative control group of healthy animals (Table 3).

The following leucocyte formula parameters are frequently clinically used for detecting systemic infection or inflammation and were analyzed to identify any potential secondary effects induced by the treatment: white blood cells count (WBC), lymphocytes % (Ly%), monocytes % (Mi%), and granulocytes % (Gr% ) are presented in Table 4. No significant changes of the leucocyte formula were noticed after the treatment with biopolymeric wound dressings containing or not the mefenamic acid.

## 4. Conclusions

Multiparticulate drug delivery systems (MDDS) consisting in the polymeric matrix based on collagen-dextran and/or microcapsules based on gelatin-CMCNa-alginate with embedded anti-inflammatory drug (FA) were developed as the local treatment for burn. These MDDS were obtained in a sponge form by freeze-drying, showed good absorbent properties, and degraded in time giving possibility to control the release of drugs. The kinetic profiles recorded a reduced burst release effect and a prolonged drug release for 48 hours for the MDDS in comparison with sponge with drug incorporated in free form. All studied MDDS displayed good biocompatibility, but in particular the matrix with 30% microcapsules and FA (M4). This also revealed the highest anti-inflammatory potential, probably due to the encapsulated FA and controlled release over 48 hours. The collagen biopolymers associated with anti-inflammatory agent accelerated the healing process with beneficial effects on the critical phases of the cicatrizing process with a faster epithelial regeneration and a minimal scarring compared to the control group. The best results were obtained with that system supported by the *in vitro* studies regarding the physical-chemical properties and resistance to enzymatic biodegradation. Besides the beneficial cicatrizing effect on experimental animals, the treatment with collagen sponges was associated with no secondary systemic or topic effects comparing to the control group which developed impaired topic inflammation process in the first days and a longer period of healing. In conclusion, this study validated novel MDDS for drug delivery in skin burn wound care, revealing a polymeric matrix base on collagen/dextran with the release of flufenamic acid in a free and encapsulated form as the most promising composition for future burn wound healing application.

## Figures and Tables

**Table 1:** Correlation coefficients for FA release from collagen spongious supports determined by application of Higuchi, Zero-order, and Power law models; kinetic parameters for Power law model; drug-released percentage.

Spongious matrices	Higuchi model	Zero-order model	Power law model	Release exponent	Kinetic constant (1/min^n^)	Drug-released percentage (%)
M1	0.9722	0.8741	0.9864	0.37	0.089	86.81^∗^
M2	0.9744	0.8566	0.9828	0.41	0.034	85.30^∗∗^
M3	0.9697	0.8430	0.9815	0.40	0.033	76.20^∗∗^
M4	0.9621	0.8264	0.9784	0.39	0.048	95.01^∗∗^
M5	0.9525	0.8041	0.9772	0.36	0.059	91.39^∗∗^

^∗^Released drug percentage after 10 hours—FA incorporated in free form; ^∗∗^released drug percentage after 48 hours—FA incorporated in multiparticulate systems.

**Table 2:** Wound diameter (mm) evolution after treatment with spongious matrices loaded with FA in experimentally induced burn to Wistar rats.

	Day 1 Mean ± SD	Day 3 Mean ± SD	Day 5 Mean ± SD	Day 7 Mean ± SD	Day 10 Mean ± SD	Day 12 Mean ± SD	Day 14 Mean ± SD	Day 17 Mean ± SD
M1	10 ± 0	9 ± 0.7^∗∗∗^	7 ± 1.9^∗∗∗^	5.2 ± 2.8^∗∗^	3.6 ± 2.9^∗∗∗^	1.4 ± 1.9^∗∗^	1 ± 1.4^∗^	0.2 ± 0.4
M2	10 ± 0	9.5 ± 0.6^∗∗∗^	8 ± 0.8	5.75 ± 2.2	4.25 ± 1.0^∗∗^	2.25 ± 1.0	0 ± 0	0 ± 0
M3	10 ± 0	10 ± 0.8^∗∗^	6.75 ± 1^∗∗∗^	6.25 ± 0.5	4.75 ± 1.0^∗^	2.25 ± 2.6	1.75 ± 2.1	0 ± 0
M4	10 ± 0	9 ± 0.1^∗∗∗^	6 ± 0.6^∗∗∗^	5.25 ± 1.2^∗^	3.75 ± 1.1^∗∗∗^	1.75 ± 0.8	1 ± 1.1	0 ± 0
M5	10 ± 0	9.25 ± 0.1^∗∗∗^	7 ± 0.6^∗∗^	6 ± 1.2	5.25 ± 1.1	0.75 ± 0.8^∗∗^	0.5 ± 1.1^∗^	0 ± 0
Control	10 ± 0	11.75 ± 0.4	10.75 ± 0.4	8.67 ± 1.2	7.83 ± 1.1	5.5 ± 0.5	2 ± 2.0	1 ± 1.4
ANOVA (P)	NS	*p* < 0.0001	*p* < 0.0001	*p* = 0.001	*p* < 0.0001	*p* < 0.005	NS	NS

SD = standard deviation, Dunnett's multiple comparison test: ^∗^*p* < 0.05, ^∗∗^*p* < 0.01, ^∗∗∗^*p* < 0.001, and ANOVA (NS = not significant at *p* > 0.05). Control = animals with burn induced but without spongious matrix treatment.

**Table 3:** The variation of the red blood cell level, hemoglobin, and erythrocyte indices after experimentally induced burns to Wistar rats and treated with biopolymeric spongious matrices loaded with FA.

	RBC (×10^6^/*μ*l) Media ± SD		HGB (g/dl) Media ± SD		HCT (%) Media ± SD		MCV (fl) Media ± SD		MCH (pg/cel) Media ± SD		MCHC (g/dl) Media ± SD		RDW Media ± SD	
M1	8.44	±0.4	15.40	±0.7	41.60	±1.0	49.33	±3.1	18.20	±0.2	36.97	±2.4	18.07	±0.4
M2	8.06	±0.5	15.03	±0.1	40.01	±1.9	49.67	±1.7	18.73	±1.2	37.67	±1.7	18.23	±0.3
M3	8.26	±0.1	15.07	±1.1	41.01	±2.4	49.33	±3.4	18.10	±0.9	36.67	±3.2	17.63	±0.5
M4	8.26	±1.1	15.87	±0.8	39.59	±4.9	48.33	±3.5	18.77	±1.6	39.23	±3.3	18.50	±0.8
M5	8.58	±0.4	15.17	±0.7	41.03	±1.8	47.67	±2.1	17.67	±1.0	37.03	±3.1	18.63	±0.2
Control	8.04	±0.4	15.47	±1.1	38.32	±2.5	47.67	±0.6	19.20	±0.5	37.32	±5.3	18.42	±0.7
Negative control	8.10	±0.8	15.43	±1.2	38.32	±2.4	47.33	±2.1	19.03	±0.6	40.17	±0.7	18.20	±0.7

RBC = red blood cell, HGB = hemoglobin, HCT = hematocrit, MCV = mean cell volume, MCH = mean cell hemoglobin, MCHC = mean cell hemoglobin concentration, RDW = red blood cell distribution width, SD = standard deviation. Control = group of animals with induced burns without spongious matrix treatment. Negative control = healthy animals.

**Table 4:** The variation of the white blood cell count after experimentally induced burns to Wistar rats and treated with biopolymeric spongious matrices.

	WBC (×10^3^/*μ*l) Media ± SD		Ly% Media ± SD		Mo% Media ± SD		Gr% Media ± SD	
M1	3.90	±0.8	55.03	±6.8	13.63	±2.6	31.30	±7.4
M2	4.41	±1.0	54.83	±7.1	15.03	±3.0	30.13	±5.8
M3	4.10	±0.4	55.30	±2.8	13.28	±1.0	31.38	±1.9
M4	4.18	±1.6	33.63	±1.2	17.23	±4.5	49.10	±1.6
M5	4.56	±1.1	45.88	±1.6	13.84	±3.1	40.27	±7.9
Control	4.04	±0.7	43.17	±1.0	14.80	±2.1	42.07	±2.9
Negative control	3.89	±0.5	40.71	±5.6	15.42	±1.7	40.28	±1.3

WBC = white blood cell, Ly% = lymphocyte %, Mo% = monocyte %, Gr% = granulocyte %, SD = standard deviation. Control = group of animals with induced burns without spongious matrix treatment. Negative control = healthy animals.

## Data Availability

The data used to support the findings of this study are included within the article.
